# Identification of a Ubiquinone–Ubiquinol Quinhydrone Complex in Bacterial Photosynthetic Membranes and Isolated Reaction Centers by Time-Resolved Infrared Spectroscopy

**DOI:** 10.3390/ijms24065233

**Published:** 2023-03-09

**Authors:** Alberto Mezzetti, Jean-François Paul, Winfried Leibl

**Affiliations:** 1 Laboratoire de Réactivité de Surface, LRS, Sorbonne Université, CNRS, 4 Place Jussieu, 75005 Paris, France; 2 Institute for Integrative Biology of the Cell (I2BC), CEA, CNRS, Université Paris-Saclay, 91198 Gif sur Yvette, France; 3 Unité de Catalyse et Chimie du Solide, Centrale Lille Université d’Artois, Université de Lille, UMR CNRS 8181-UCCS, 59000 Lille, France

**Keywords:** quinhydrone, ubiquinone, ubiquinol, charge-transfer complex, Fourier Transform Infrared (FTIR) difference spectroscopy, rapid-scan FTIR, bacterial reaction center (RC), chromatophores, *Rhodobacter (Rb.) sphaeroides*

## Abstract

Ubiquinone redox chemistry is of fundamental importance in biochemistry, notably in bioenergetics. The bi-electronic reduction of ubiquinone to ubiquinol has been widely studied, including by Fourier transform infrared (FTIR) difference spectroscopy, in several systems. In this paper, we have recorded static and time-resolved FTIR difference spectra reflecting light-induced ubiquinone reduction to ubiquinol in bacterial photosynthetic membranes and in detergent-isolated photosynthetic bacterial reaction centers. We found compelling evidence that in both systems under strong light illumination—and also in detergent-isolated reaction centers after two saturating flashes—a ubiquinone–ubiquinol charge-transfer quinhydrone complex, characterized by a characteristic band at ~1565 cm^−1^, can be formed. Quantum chemistry calculations confirmed that such a band is due to formation of a quinhydrone complex. We propose that the formation of such a complex takes place when Q and QH_2_ are forced, by spatial constraints, to share a common limited space as, for instance, in detergent micelles, or when an incoming quinone from the pool meets, in the channel for quinone/quinol exchange at the Q_B_ site, a quinol coming out. This latter situation can take place both in isolated and membrane bound reaction centers Possible consequences of the formation of this charge-transfer complex under physiological conditions are discussed.

## 1. Introduction

**Figure 1:**
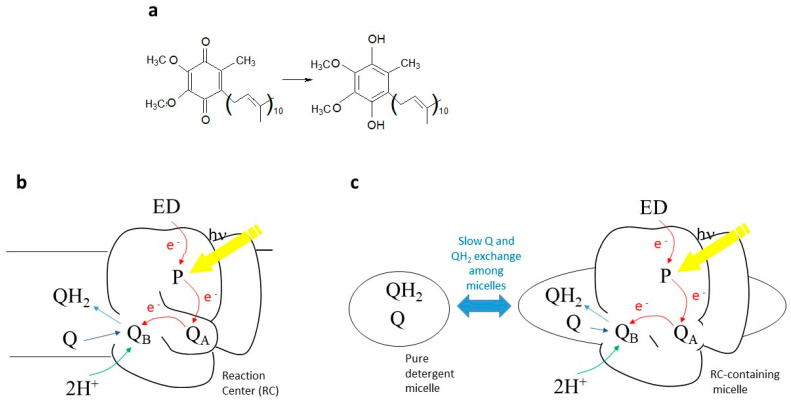
(**a**) Ubiquinone structural formula and its reduction to ubiquinol. *(***b**) Scheme of ubiquinone photoreduction in photosynthetic membranes. After 2 consecutive series of photoinduced electron transfer reactions, the doubly reduced Q_B_H_2_ leaves the RC and is replaced by a new Q molecule coming from the quinone pool in the membrane. (**c**) Scheme if ubiquinone photoreduction in detergent-isolated photosynthetic reaction centers (RCs). In this case, after 2 consecutive series of photoinduced electron transfer reactions, the doubly reduced Q_B_H_2_ leaves the RC to go into the detergent phase of the RC-containing detergent micelle. A Q/QH_2_ exchange at the Q_B_ site also takes place in this case. However, here the situation is complicated by the Q/QH_2_ exchange between RC-containing detergent micelles and pure detergent micelles. P stands for the primary donor (dimer of BChl *a* molecules); ED stands for a generic electron donor, in the case of the experiments presented in this manuscript 2,3,5,6-tetramethyl-p-phenylenediamine (diaminodurene DAD).

Quinones are key players in bioenergetic processes such as respiration and photosynthesis [1]. They work as electron carriers between membrane proteins, and the Q → QH_2_ transition couples electron transfer to the formation of a transmembrane proton gradient. In photosynthesis, the reduction of Q to QH_2_ is catalyzed by light and takes place in reaction centers (RCs), which are integral membrane proteins. The reduction can be triggered by actinic light and makes photosynthetic RCs and membranes ideal model systems to investigate general principles governing the bioenergetic and structural roles of quinones (in oxidized and reduced form) in biochemistry, also outside of photosynthesis. In RCs from purple photosynthetic bacteria, sequential absorption of two photons by a special couple of bacteriochlorophylls, the primary electron donor P, leads to the double reduction and protonation of a weakly bound ubiquinone, named Q_B_, to ubiquinol Q_B_H_2_ [2] (see Figure 1a). The ubiquinol thus formed is released from its binding site into the membrane and replaced by an oxidized ubiquinone from the quinone pool in the membrane (see Figure 1b). The ubiquinol QH_2_ is then re-oxidized by another membrane enzyme of the photosynthetic electron-transfer chain, the cytochrome bc_1_ complex, which acts as a ubiquinol oxidase/cytochrome reductase [3]. Water-soluble cytochrome c shuttles between the RC and the bc1 complex to close the electron transfer cycle.

The cyclic mechanism of the above-mentioned redox reactions acts as a light-driven proton pumping system, as it moves protons from the cytoplasm to the periplasm. The resultant H^+^ gradient is used for the synthesis of ATP. The light-induced formation and release of Q_B_H_2_ from the RC binding pocket and its replacement by an oxidized quinone has been characterized by several techniques, among which Fourier Transform Infrared (FTIR) difference spectroscopy [4,5] occupies a special role ([6,7,8,9] and refs. therein). Indeed, this technique not only allows monitoring of the change in redox state of quinones; it also makes it possible to visualize interaction of the quinones with the surrounding protein binding pocket, as well as molecular events associated with quinone reduction (localized protein conformational changes, protonation of amino acid side chains, etc.) [4]. Time-resolved FTIR difference spectroscopy [10] is even more powerful, as it allows these processes to be followed in time. The technique has found widespread application in photosynthesis (see [11,12] for recent reviews). Notably, it has been applied to the study of the reduction of the Q pool in the native chromatophore membrane (see Figure 1b) and in biomimetic membranes upon multiple turnovers of electron transfer, allowing researchers to directly monitor the process through infrared marker bands for Q and QH_2_ ([7,8,13,14]). Associated events such as different interaction of Q and QH_2_ with the headgroups of the membrane phospholipids [13] or conformational changes of other membrane proteins could also be studied [7,13,14]. Multiple reduction of quinones in detergent-isolated RCs (in the presence of a five-fold excess of exogeneous quinone) was also reported [6], but it was complicated by the existence of Q and QH_2_ exchanges between pure detergent micelles and RC-containing detergent micelles (see Figure 1c).

Detailed chemometric investigation of time-resolved IR difference spectra in isolated RCs (see Figure 1c) and chromatophores from *Rhodobacter (Rb). sphaeroides* R26 (now classified as *Cereibacter sphaeroides*) have put in evidence that under continuous illumination a peculiar form of reduced ubiquinone can be formed [7,15]. In the present paper, we show that this new form can also be produced after two flashes in isolated RCs. Furthermore, comparison of experiments in D_2_O and H_2_O, along with quantum chemistry calculations, provide evidence that the new form is a charge-transfer ubiquinone–ubiquinol quinhydrone complex. Whereas quinhydrone complexes from simpler quinones have been characterized in several papers ([16,17,18,19,20,21] and refs. therein) and were reported to have a possibly important chemical or biochemical role [18,19], in the case of ubiquinol–ubiquinone charge-transfer complex, only a few reports exist. It was assumed to act as a cofactor in bacterial disulfide bond formation [22] and its existence has been postulated at the Q_O_ site of cytochrome bc_1_ complex [23,24], as well as in artificial phospholipid bilayers [25]. In addition, ubiquinone-0 and ubiquinol-0 were found to form a quinhydrone complex in EtOH [22].

Here, on the basis of the presented experimental data, we propose that this ubiquinone–ubiquinol charge-transfer quinhydrone complex forms in photosynthetic membranes, RC-containing detergent micelles and/or at specific binding pockets of RCs under specific conditions where oxidized and reduced forms of ubiquinone are present in a confined environment. Ubiquinone redox chemistry is particularly complicated [26], and the possible role of quinhydrone complexes in electron transfer reactions in membranes has already been proposed [25]. On the basis of the results presented here, the formation of ubiquinol–ubiquinone charge transfer complexes—including in other kinds of membranes, e.g., mitochondrial ones, or in specific environments—should be considered and might be of peculiar biochemical relevance.

## 2. Results

### 2.1. Light-Induced Fourier Transform Infrared (FTIR) Difference Spectroscopy on Chromatophores

**Figure 2:**
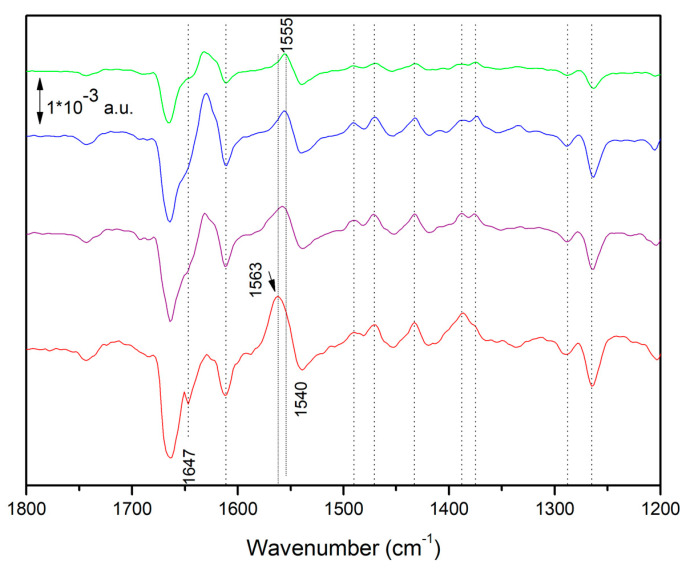
Fourier Transform Infrared (FTIR) difference spectra on *Rhoodobacter (Rb.) sphaeroides* chromatophores recorded under illumination with different intensities, between 2 and 4 s after the onset of light exposure. The relative light intensity is (top to bottom) 1 (green):2 (blue):5 (purple):8 (red). Spectra are recorded on the same sample; measurement times (both in terms of light exposure and number of measurement cycles) are minimized in order to have negligeable light-induced sample degradation or fatigue. Experiments repeated on other samples provided similar results. Peak position should be considered to have a ±1 cm^−1^ uncertainty.

Figure 2 shows FTIR difference spectra on chromatophores recorded between 2 and 4 s of illumination at different light intensities in the presence of diaminodurene (DAD) acting as electron donor. DAD ensures fast reduction of the photo-oxidized primary donor P^+^ [27], thereby making possible a double reduction of the ubiquinone in the Q_B_ site of the RCs and its replacement by an oxidized Q from the ubiquinone pool. Under sufficiently long periods of light exposure, the reduction of several quinones (from the pool) per RC is possible. The presence of specific inhibitors blocks QH_2_ reoxidation by the bc_1_ complex. The overall shape of the difference spectra is in agreement with previous reports [7,13,14], including spectra obtained in chromatophores from other species [9]. Interestingly, a difference between spectra recorded under low illumination intensity and those recorded at higher intensity is clearly visible. A positive band at ~1563 cm^−1^ is present only under high light intensity, as reported before [7,14]. The band is particularly strong in the spectrum recorded under the highest intensity.

**Figure 3:**
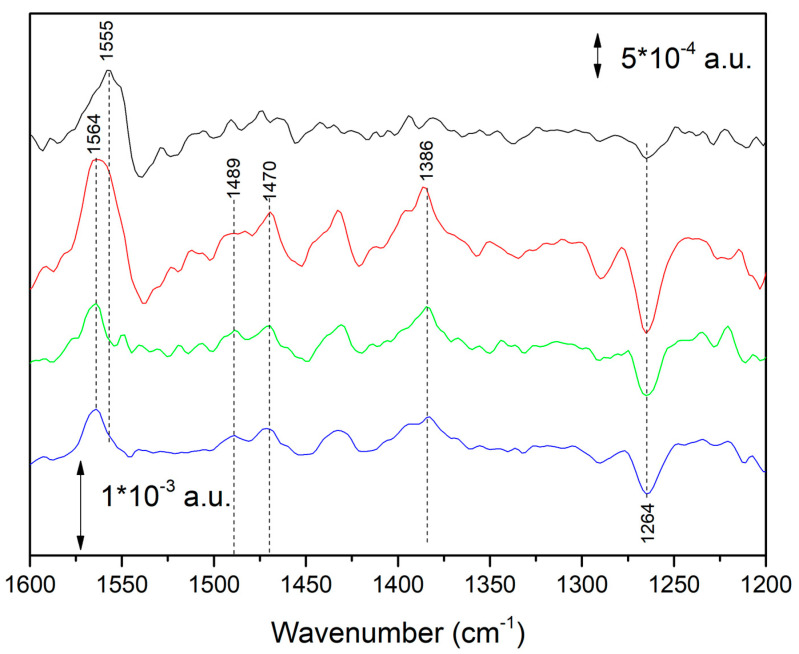
Time evolution of the FTIR difference spectra of *Rb. sphaeroides* chromatophores after onset of continuous illumination at higher illumination intensity (upper two traces) and after switching off the illumination (two traces at the bottom). Black trace (first from top): recorded between 0 and 434 ms after light onset. Red trace (second from top): recorded between 3878 and 4312 ms after light onset. Green trace (third from top): recorded between 434 and 868 ms after switching off the light. Blue trace (fourth from top) recorded between 1300 and 3000 ms after switching off the light. Peak position should be considered to have a ±1 cm^−1^ uncertainty.

Figure 3 shows the time evolution under (and after) illumination at higher intensity. Interestingly, the shape of spectra changes with time both under and after illumination. At least three events are taking place, so that three spectral components are present. The first component is characterized by the strong positive band at 1555 cm^−1^; the second component is characterized by the negative band at 1264 cm^−1^; the third component is characterized by the positive band at 1563 cm^−1^. A detailed kinetic investigation (using Multivariate curve Resolution strategies) of the different spectral components, under the very same conditions, has already been reported: one component was found to essentially reflect a protein conformational change, whereas two components where both found to reflect a reduction of the ubiquinone pool [7].

In the present paper, we focus our attention on the time-resolved FTIR difference spectrum recorded soon after switching off of the intense illumination (green trace in Figure 3, recorded between 434 and 868 ms after the end of the illumination). The strong 1555 (+)/1540 (−) cm^−1^ band that was present in the central part of the spectra under illumination disappeared, whereas a positive band at ~1564 cm^−1^ remained. The previous chemometric analysis of time-resolved FTIR difference spectra under these conditions has indeed shown that a spectral component reflecting ubiquinone pool reduction characterized by this positive band at ~1564 cm^−1^ exists when the sample is exposed to high illumination intensity, and that after illumination its decay is slower than the one of the component responsible of the 1555 (+)/1540 (−) cm^−1^ band [7]. The same analysis also showed that the species reflecting ubiquinone pool reduction and characterized by the ~1564 cm^−1^ band is also overwhelmingly dominant under strong illumination conditions and at early times (1–3 s) after the end of the illumination period. This means that the two spectra in Figure 3 recorded after the end of the illumination essentially reflect this spectral component. 

**Figure 4:**
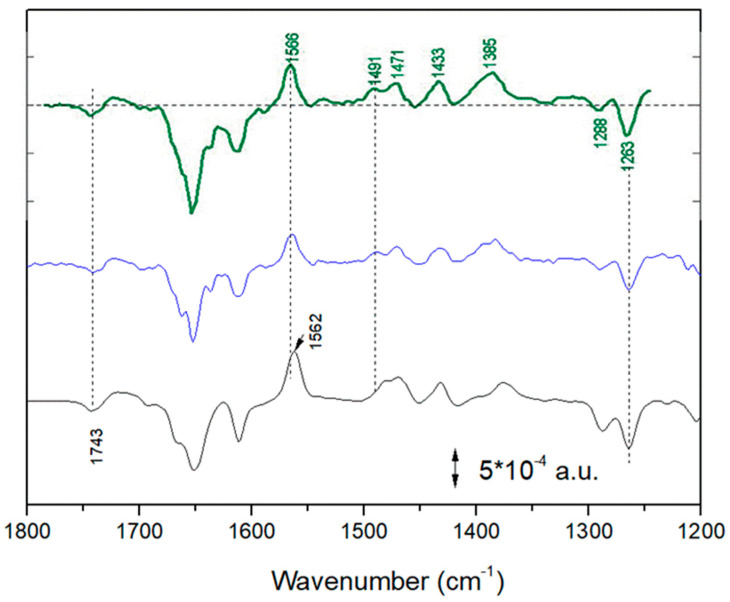
FTIR difference spectra reflecting reduction of the quinone pool with formation of the new species characterized by a band at ~1565 cm^−1^. From top to bottom: green spectrum, taken from [7], obtained after a Multivariate Curve Resolution treatment of time-resolved FTIR difference data on chromatophores. Blue spectrum, recorded between 1, 3 and 3 s after strong illumination (last bottom trace of time-resolved FTIR spectra shown in Figure 3). Black spectrum: spectrum recorded on chromatophores between 1 and 3 s after strong illumination in D_2_O. Peak position should be considered to have a ±1 cm^−1^ uncertainty. Green spectrum was adapted with permission from Ref. [7], Copyright 2009 American Chemical Society.

To better understand the nature of this spectral component, time-resolved FTIR spectra were also recorded in D_2_O. Spectra obtained soon after the illumination period in H_2_O and D_2_O are compared in Figure 4.

Small spectral shifts were observed in the 1500–1200 cm^−1^ region, in partial agreement with a previous report obtained under milder illumination conditions [13]. In the present paper we focus our attention on the very small downshift of the ~1564/1565 cm^−1^ band upon D/H exchange (in D_2_O it absorbs at 1562 cm^−1^). This small isotopic effect is not the behavior one would expect for an amide II band or any other band reflecting—even partially—a OH or NH vibration. 

We now put together all the available pieces of information obtained so far on chromatophores.

(1) A spectral component, characterized by a strong ~1564 cm^−1^ positive peak, is present only under a strong illumination condition of chromatophores;
(2) A previous detailed chemometric analysis [7] demonstrated that this spectral component represents a reduction of (a fraction of) the ubiquinone pool;
(3) The most characteristic band of this spectral component, the ~1564 (+) cm^−1^ peak, shows only a small downshift (~2 cm^−1^) upon D/H exchange.

### 2.2. Light-Induced FTIR Difference Spectroscopy on Isolated Reaction Centers

**Figure 5:**
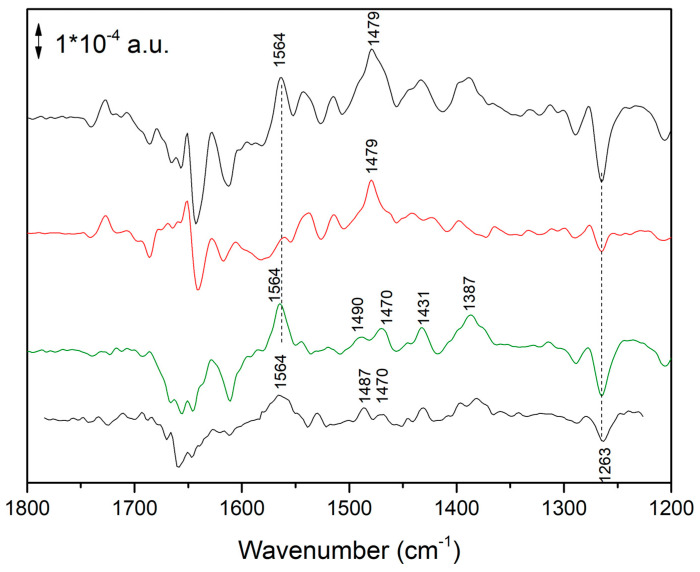
FTIR difference spectra recorded after one (red trace—2nd from top) and two flashes (black trace—1st from top). The difference spectrum after 2 flashes represents a mixture of ~75% QH_2_ state and of 25% Q_B_^−^ state [6]. The third trace from the top (green) was obtained by subtracting (multiplied by a 0.3 coefficient) a Q_B_^−^/Q_B_ difference spectrum (red—2nd trace from the top) from the difference spectrum obtained after two flashes (black—1st trace from the top). This third trace represents the species formed after a double reduction of Q_B_ and shows an intense positive 1564 cm^−1^ band. The fourth trace from the top (black) was taken from [15], obtained after a chemometric analysis of time-resolved FTIR difference spectra of isolated RCs under and after continuous illumination, where four spectral contributions were identified. The plotted trace—characterized by a strong positive peak at 1564 cm^−1^—is believed to represent a double-reduced form of Q [15]. The last trace from the top (black) was reprinted by permission from Ref. [15], Copyright 2011, Springer.

In Figure 5, FTIR difference spectra recorded after one and two flashes on isolated RCs from *Rb. sphaeroides* are shown (second and first trace from the top, respectively). It has previously been shown that after the first flash the semiquinone Q_B_^−^ is formed in nearly all the RCs. Conversely, after the second flash, a Q_B_H_2_ (or more properly said a QH_2_, considering that the formed ubiquinol leaves the protein binding pocket) is formed in ~75% of the RCs [6]. The obtained difference spectrum therefore represents a mixture of ~75% QH_2_ state and 25% Q_B_^−^ state. As previously reported, by subtracting a Q_B_^−^/Q_B_ spectrum (multiplied by an adequate coefficient between 0.2 and 0.3) from the spectrum recorded after two consecutive flashes a “pure” spectrum reflecting ubiquinol formation can be obtained. This operation was limited in our previous paper [6] to the 1500–1200 cm^−1^ spectral region. Here, we report that when this operation is performed on a larger spectral range, the characteristic quinhydrone charge-transfer band at ~1564 cm^−1^ appears (Figure 5, third trace from top).

It is important to notice that a band—albeit broader—at the same position was present in one of the four IR difference spectra calculated by a chemometric approach (Multivariate Curve Resolution) on a series of time-resolved FTIR difference spectroscopy experiments carried out under and after continuous illumination at different powers of illumination [15]. The spectrum is reported as the lower trace in Figure 5. 

### 2.3. Comparison between Experiments in Different Conditions

**Figure 6:**
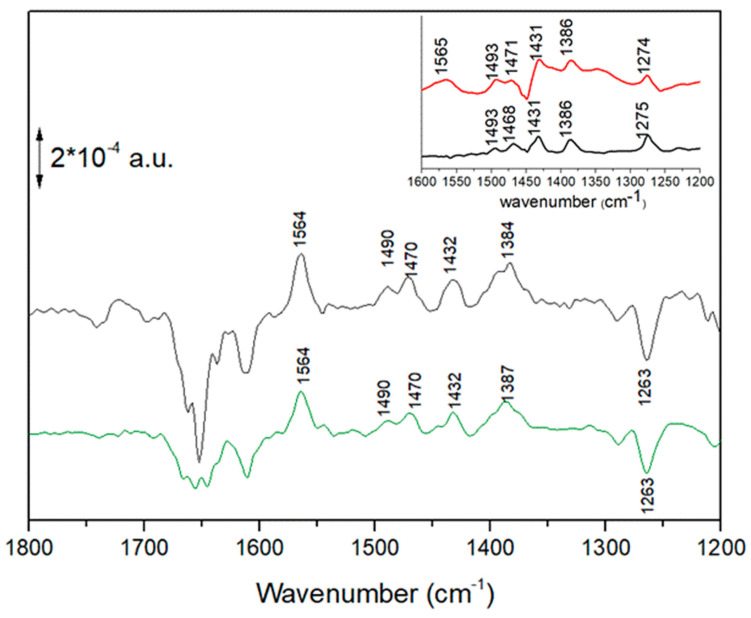
Upper trace, black: FTIR difference spectrum recorded on chromatophores between 1302 and 1736 ms after a 4.3 s period of intense illumination (see also Figure 3). Lower trace (green): calculated FTIR spectrum in detergent-isolated RCs after two saturation flashes (see also Figure 5). Inset: spectra of synthetic Q_6_H_2_ in cyclohexane in freshly prepared solution (black trace) and after exposure of the solution to air for some hours (red trace).

In Figure 6, the FTIR difference spectrum measured on chromatophores at early times after a 4 s period of intense illumination (see also Figure 3) and the calculated FTIR spectrum reflecting in detergent-isolated RCs (see also Figure 5) are compared. In the 1600–1200 cm^−1^ region the similarity is striking, especially for the positive 1564 cm^−1^ and the negative band at 1263 cm^−1^ (characteristic of Q disappearance) [13]. 

In the inset of Figure 6, spectra of synthetic Q_6_H_2_ in cyclohexane in freshly prepared solution (black trace) and after exposure of the solution to air for some hours (red trace) are shown. Solvent contributions were subtracted. It appears evident that after exposure to air a broad 1565 cm^−1^ band is formed. We interpreted this band as arising from a quinhydrone complex in solution, the result of the association of the formed ubiquinone Q_6_ (produced by the Q_6_H_2_ molecules, which were oxidized by O_2_ in the air) and of the Q_6_H_2_ (still present in high concentration in the solution). Spontaneous association of oxidized and the reduced form of ubiquinone to form a quinhydrone complex was already reported for Q_0_ and Q_0_H_2_ in EtOH [22]. It is known that the formation of quinhydrone complexes entails the formation of new bands in the 1700–1400 cm^−1^ region [16,17,18,19,20,21].

In all spectra (chromatophores at early times after illumination, isolated RCs, air-exposed cyclohexane solution of Q_6_H_2_), this band is present at the same position. Furthermore, this band is always associated with bands at 1490, 1470, ~1432 and 1384–1387 cm^−1^, characteristic of ubiquinol [7,13,28,29]. We took this as evidence of the formation of the same molecular species, a quinhydrone complex, in all three different conditions. This was confirmed by theoretical calculations (see below).

### 2.4. Theoretical Calculations

**Figure 7:**
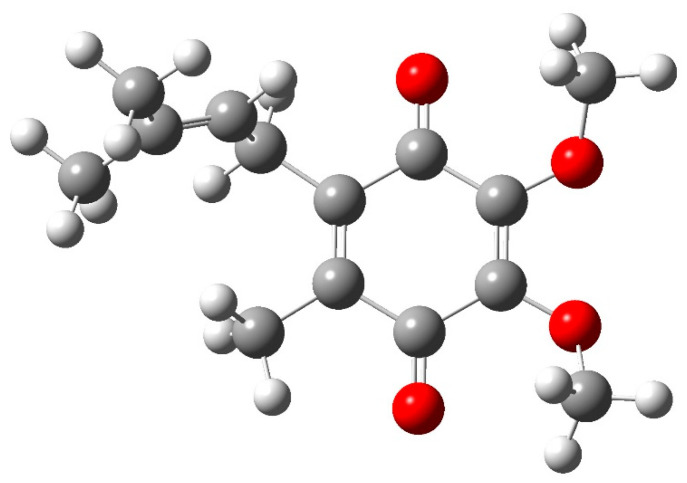
Model quinone molecule used for theoretical calculations of IR frequencies.

DFT (B3LYP) [30] calculations were performed on model molecules to confirm the assignment of the ~1565 cm^−1^ band to the formation of quinhydrone complexes. The model molecule used to mimic ubiquinone-10 (and ubiquinone-6) is represented in Figure 7. The methoxy groups and the methyl group were added on the benzene ring to mimic the real model, but the lateral chain was reduced to reduce calculation time. 

**Figure 8:**
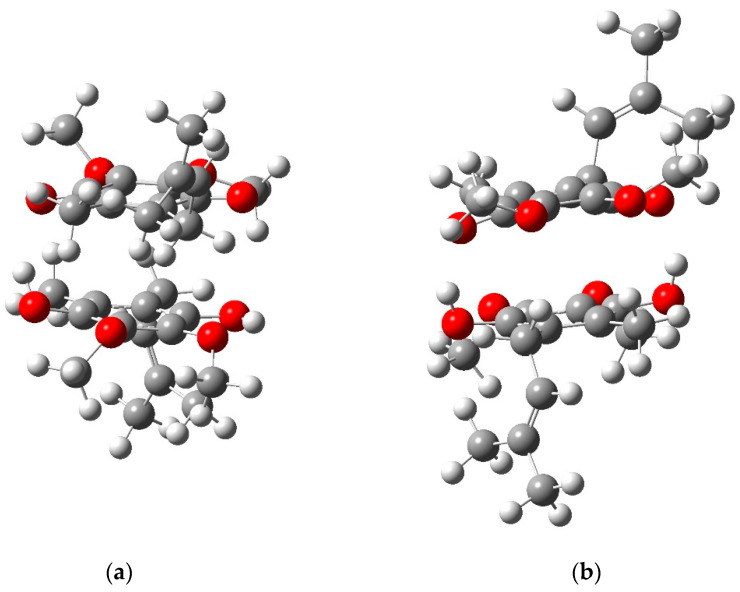
Stable quinone–quinol quinhydrone complex after optimizations. (**a**) Most stable geometry (∆_r_E = 106 kJ mol^−1^); (**b**) stable complex with two hydrogen bonds (∆_r_E = 90 kJ mol^−1^).

Once one of the quinone molecules was reduced, the formation of the dimer complex was an exothermic reaction ∆_r_E = 106 kJ mol^−1^. The two molecules can form one or two hydrogen bonds. The two most stable geometries are represented in Figure 8.

One of the protons can be transferred from QH_2_ to Q, but the new system is less stable than the previous ones (∆_r_E = 37 kJ mol^−1^). 

The calculations of the IR spectra for both the reactants (Q and QH_2_) and the quinhydrone complex demonstrate the apparition of a new intense band 46 cm^−1^ below the intense unscaled band of isolated ubiquinone computed at 1700 cm^−1^. This new band is caused by the coupling of antisymmetric elongation of the quinone C=O bonds with the rocking of the OH bond of the QH2 molecule. This band is mainly localized on the quinone and its frequency is almost unchanged by D/H exchange. Taking a typical scaling factor of 0.9668, we obtained a value of 1599 cm^−1^, in good agreement with the ~1564 cm^−1^ experimental value. The absence of a significant downshift upon D/H exchange is in nice agreement with our experimental observations on the spectra recorded at early times after a 4 s period of intense illumination, strengthening the proposed formation of a quinhydrone complex under these conditions.

## 3. Discussion

It is well-known that quinones and hydroquinones have a general tendency to associate to form a charge-transfer state named quinhydrone. Quinhydrone formation is known to entail the formation of new IR bands, absent from quinone and quinol IR spectra [16,17,18,19,20,21].

Until now, just one clear evidence (from UV-Vis spectroscopy) was reported that this charge-transfer complex can be formed, in EtOH solution, by ubiquinone/ubiquinol without isoprenoid chain, i.e., Q_0_ and Q_0_H_2_ [22]. In this paper, we show that under specific conditions in photosynthetic membranes and isolated RCs this charge transfer complex can also be formed by ubiquinone and ubiquinol endowed with a long isoprenoid chain. We found that such a complex also forms in solution of Q_6_H_2_ in cyclohexane exposed to air. It is interesting to notice that the transient formation of quinhydrone was reported during chemical reduction of ubiquinone-10 (Q_10_) [31].

**Figure 9:**
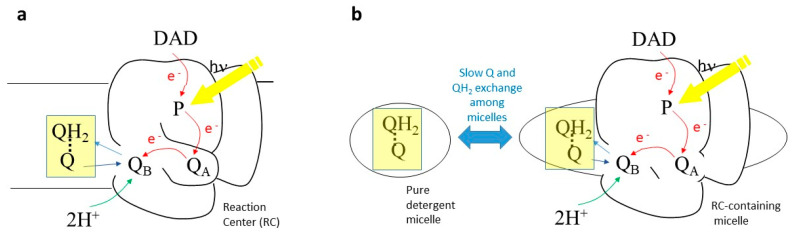
Conditions under which quinhydrone complex could form in chromatophores (**a**) or detergent-isolated RCs (**b**).

Let us consider two peculiar situations: in bacterial photosynthetic membranes under strong light, a large number of ubiquinone molecules from the Q pool can be reduced to QH_2_ per RC. The absence of intense characteristic bands due to the oxidized primary donor P+ is direct evidence that external redox donors are able to rapidly reduce P+, so that the very same P dimer is ready to absorb a new photon a few milliseconds after the previous one. The problem of “keeping the pace” imposed by the light intensity is more on the acceptor side of the RC (it should be kept in mind that in the present experimental conditions the natural re-oxidation of QH_2_ by the bc_1_ complex is blocked). As a possible hypothesis, we can speculate that quinhydrone complex formation under high light conditions derives from the Q_B_H_2_, leaving the Q_B_ site, which meets an oxidized Q molecule from the pool (see Figure 9a). The high intensity of the quinhydrone bands suggests that complex formation is not limited to the entrance of the Q_B_ pocket but, more likely, is preserved also in the membrane due to a reduced mobility of Q and QH_2_ in *Rb. sphaeroides* membranes. Indeed, Woronowicz et al. [32] already suggested that the packing density of LH2 antenna complexes significantly slows down the diffusion of Q redox species between the RC and the bc_1_ complex. In this interpretation, quinhydrone complex formation is a consequence of spatial constraints that force ubiquinone and ubiquinol to share a limited space. 

Let us now examine the second situation. It is well-known that in isolated RCs long chain ubiquinone molecules exist only in detergent micelles, which can be either RC-containing or pure detergent micelles [6,33]. In both kind of micelles, space is limited and this fact most probably favors the association of Q and QH_2_ and the formation of charge-transfer quinhydrone complexes (see Figure 9b). 

The fact that formation of quinhydrone complexes was never reported to date either in chromatophores or in isolated RCs is most probably due to the simultaneous presence of several pigments, which hampers detection of the characteristic Vis band of quinhydrones in the 500–600 nm region [34]. UV-Vis spectroscopy is widely used in biochemistry, but detection of an electronic band arising from quinhydrone in samples full of other chromophores should therefore not be considered as surprising, given the high ε of carotenoids, bacteriochlorophylls and bacteriopheophytins, among others. Indeed, our attempts to measure quinhydrone electronic bands by UV-Vis spectroscopy did not provide any precise answer. It should also be noticed that the only reported UV-Vis spectrum of a ubiquinone–ubiquinol quinhydrone complex in EtOH solution [22] suggests that the characteristic electronic transition at ~500 nm has a quite low extinction coefficient (~100 L mol^−1^ cm^−1^).

It was already suggested that ubiquinone–ubiquinol quinhydrone complexes might be stable in biological membranes [25]. With our FTIR study we confirm that formation of such charge transfer complexes is possible in bacterial photosynthetic membranes and possibly at the Q_B_ site (or close to the Q_B_ site) in bacterial RCs. The presence of quinhydrone is possibly related to the complicated antioxidant/prooxidant chemistry of ubiquinone in mitochondrial membranes [35]. Further studies (in membranes and in model environments) are required to better characterize a possible biochemical and biological role of ubiquinone–ubiquinol quinhydrone complexes in biological membranes.

## 4. Materials and Methods

### 4.1. Sample Preparation

The photosynthetic bacterium *Rb. sphaeroides* R26 was grown as described in [36]. Chromatophores and isolated RCs from *Rb. sphaeroides* R26 were obtained as described in [37,38]. Samples for time-resolved FTIR spectroscopy were prepared as follows. In the case of chromatophores, samples for FTIR measurements were prepared by diluting the chromatophores to a concentration of 3 µM in RCs in 70 mM Tris buffer (pH 8) and adding 10 µM antimycin and 10 µM myxothiazol (specific inhibitors of the cytochrome bc_1_ complex). To avoid spectral contributions from the photo-oxidation of the primary donor P, 10 mM sodium ascorbate and 20 mM 2,3,5,6-tetramethyl-p-phenylenediamine (diaminodurene DAD) were used to ensure fast reduction of P^+^ [13,27]. The suspension was centrifuged at 220,000× *g* for 15 min. The obtained pellet was squeezed and then sealed between two CaF_2_ windows in order to obtain an absorbance in the amide I region between 0.7 and 0.9 a.u. Samples in D_2_O were prepared by diluting chromatophores in D_2_O and adding inhibitors and redox components in D_2_O. Two centrifugations and resuspensions of the pellet in D_2_O were carried out. This procedure provides deuteration of about 60% peptide NH groups of the proteins in the membrane. 

In the case of isolated RCs, they were deposited on a CaF_2_ window and dried under argon. Before complete dryness, the RC film was rehydrated with Tris buffer (pH 8, 100 mM). In order to maximize occupancy of the Q_B_ site, a fivefold excess of ubiquinone-6 (UQ6, Sigma, St. Louis, MO, USA) was added to the RC suspension. Sodium ascorbate (10 mM) and DAD (20 mM) were added as external redox components (to rapidly reduce P^+^, as in the case of chromatophores). A second CaF_2_ window was used to squeeze the sample in order to yield an absorbance in the amide I region of the spectrum of 0.6–0.9 a.u. Experiments in D_2_O were not possible in isolated RCs, as at the temperature of the experiments (281 K) P^+^ reduction by DAD is severely slowed down [6,39], and this hampers performing rapid-scan FTIR experiments with the appropriate timing (see below). Conversely, experiments in chromatophores at the same temperature were possible in D_2_O. This is probably due to the lipophilic nature of DAD.

Synthetic ubiquinol-6 was obtained by chemical reduction of ubiquinone-6 (Sigma Aldrich, St. Louis, MO, USA) by NaBH_4_ following the procedure described in [40].

### 4.2. FTIR Spectroscopy

#### 4.2.1. FTIR Spectra in Cyclohexane

Static FTIR spectra of ubiquinol-6 in cyclohexane (10^−4^ M) were recorded using an ATR module mounted on a Bruker IFS 88 spectrometer. ATR-FTIR spectra were recorded before and after 5 h exposure of the solution to air. A cyclohexane FTIR spectrum (recorded on the same ATR module) was subtracted using OPUS (OS/2) software.

#### 4.2.2. Time-Resolved FTIR Experiments under and after Continuous Illumination in Chromatophores

For light-induced FTIR difference spectra, a Bruker IFS88 FTIR spectrometer equipped with a photoconductive MCT-A detector and with OPUS software was used in the transmission mode. Measurements were performed at 281 ± 1 K using a temperature-controlled N_2_ cryostat (Oxford Instruments, Oxford, UK). 

Time-resolved FTIR spectra were recorded under and after continuous illumination of the sample with a 250 W tungsten-halogen lamp, using rapid-scan conditions adapted from those described in [7] (see also Figure S1 in supplementary information). Different experiment cycles, each with a different power of light, were used on the same sample. Conditions (temperature, number of repetitions, etc.) were chosen in order to minimize ageing and photoaging effects on the sample. Experiments were then repeated on another 3 samples, showing consistent results and high reproducibility in spectral shape (band position was found to coincide within 1–2 cm^−1^; band intensity was found to be reproducible within 4% of incertitude). Kinetics of bands growth and disappearance were also consistent within the 4 samples. 

Whereas the geometry of the sample compartment makes it difficult to quantify absolute light intensity impinging on the sample on each measurement cycle, the use of optical filters enables the quantification of the ratio of the light intensity between the different cycles. Therefore, the light intensity in the last cycle was 8-fold compared with the one of the first cycle. Intermediate cycles had a 2-fold and a 5-fold higher light intensity compared with the first cycle. 

Typically, an experimental cycle started with the recording and averaging of 200 reference interferograms in the dark (duration: 8 s). Then, the light was switched on using a mechanical shutter and interferograms were recorded and averaged in groups of 10 (duration: 434 ms). After 4.3 s the light was switched off and interferograms were recorded and averaged in groups of 10 to monitor the ‘relaxation’ of the sample. FTIR difference spectra at various times under or after the continuous illumination were calculated from the single beam spectrum (obtained from the Fourier transform of averaged interferograms), recorded in the dark before the onset of the light and the single beam spectrum obtained at time t. For each cycle, the results from several hundreds of repetitions of the illumination scheme were averaged. Between repetitions, a delay time of a few minutes was set to allow a complete relaxation of the system.

#### 4.2.3. Two-Flash Rapid-Scan FTIR Experiments on Isolated RCs

Rapid-scan FTIR measurements were performed using the experimental parameters as described in [6] (see also Figure S2 in supplementary information). The photoreactions were triggered by saturating flashes from a frequency-doubled Nd:YAG laser (7 ns, approximately 20 mJ, Quantel, France). The experimental scheme was the following. A total of 40 interferograms were recorded in the dark and averaged. Then, a first laser flash was fired and two successive interferograms were recorded to monitor the time evolution of the system. A second flash was fired and five interferograms were recorded at increasing delays to monitor the process of ubiquinol formation. Subsequent interferograms were averaged in groups of 5. The results from 4000 cycles (obtained on 4 different samples) were averaged to improve the signal-to-noise ratio. Between cycles, an appropriate delay time (3 min) was set to allow a complete relaxation of the system.

Experiments were repeated on 4 different samples, showing high reproducibility in spectral shape (band position was found to coincide within 1 cm^−1^; band intensity was found to be reproducible within 2% of incertitude). 

### 4.3. Theoretical Calculations

All the calculations were made using Gaussian 16 [41]. DFT (B3LYP) [30] calculations, including empirical dispersion corrections (D3) [42] were performed on model molecules to confirm the assignment of the new band to the formation of ubiquinone-like complexes. All the molecules and complexes were fully optimized without any constraint. A large basis set (6–311 g), [43] with polarization function on all the atoms, was used to limit BBSE correction and allow a good description of the hydrogen bonds in the ubiquinone complex.

## 5. Conclusions

In this paper, we provide clear evidence that ubiquinone–ubiquinol quinhydrone complexes can be formed in photosynthetic bacterial membranes and in detergent-isolated RCs under conditions where a large part of the quinone pool becomes reduced. We suggest that quinhydrone forms when ubiquinone and ubiquinol are forced by spatial constraints to come in close contact. The chemical and biological consequences of the formation of quinhydrone have yet to be investigated. Given the relevance of ubiquinone and ubiquinol in bioenergetics, and of ubiquinol as an anti-oxidant agent, ubiquinone/ubiquinol quinhydrone complex formation should also be explored in other systems, e.g., mitochondrial membranes. 

## Data Availability

All data are available upon request to the authors.

## Supplementary Materials

The supporting information can be downloaded at: https://www.mdpi.com/article/10.3390/ijms24065233/s1.
