# Supplementary material for: Identification of a Ubiquinone–Ubiquinol Quinhydrone Complex in Bacterial Photosynthetic Membranes and Isolated Reaction Centers by Time-Resolved Infrared Spectroscopy
Source: Int J Mol Sci. 2023 Mar 9;24(6):5233. doi: 10.3390/ijms24065233 (PMC10049466; doi:10.3390/ijms24065233)
Supplement: Supplementary file 1 [file ijms-24-05233-s001.zip › ijms-2072818-supplementary.pdf]

Electronic Supplementary information

Identification of a Ubiquinone–Ubiquinol Quinhydrone Complex in Bacterial Photosynthetic Membranes and Isolated Reaction Centers by Time-Resolved Infrared Spectroscopy

Alberto Mezzetti [1,2,*], Jean-François Paul [3] and Winfried Leibl [2]

[1] Laboratoire de Réactivité de Surface, LRS, Sorbonne Université, CNRS, 4 Place Jussieu, 75005 Paris, France
[2] Institute for Integrative Biology of the Cell (I2BC), CEA, CNRS, Université Paris-Saclay, 91198 Gif sur Yvette, France
[3] Unité de Catalyse et Chimie du Solide, Centrale Lille Université d'Artois, Université de Lille, UMR CNRS 8181-UCCS, 59000 Lille, France
* Correspondence: alberto.mezzetti@sorbonne-universite.fr

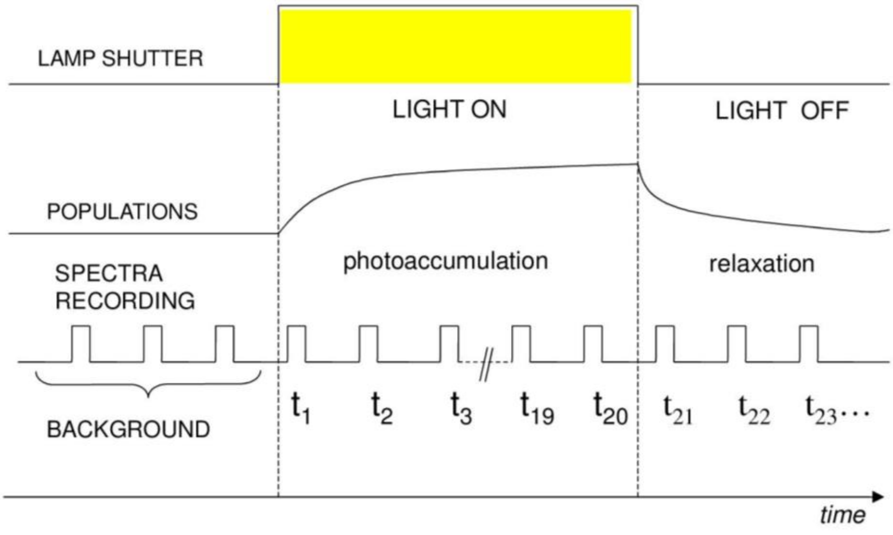

Figure S1. Scheme of experiments under and after illumination on chromatophores

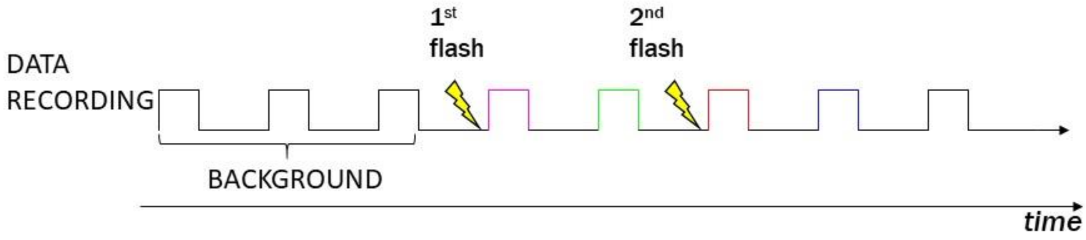

Figure S2. Scheme of experiments with two consecutuive flashes on isolated Reaction Centers.
